# Fecal DNA isolation and degradation in clam *Cyclina sinensis*: noninvasive DNA isolation for conservation and genetic assessment

**DOI:** 10.1186/s12896-019-0595-6

**Published:** 2019-12-19

**Authors:** Min Zhang, Min Wei, Zhiguo Dong, Haibao Duan, Shuang Mao, Senlei Feng, Wenqian Li, Zepeng Sun, Jiawei Li, Kanglu Yan, Hao Liu, Xueping Meng, Hongxing Ge

**Affiliations:** 1 Jiangsu Key Laboratory of Marine Biotechnology, Jiangsu Ocean University, Lianyungang, 222005 Jiangsu China; 2 Co-Innovation Center of Jiangsu Marine Bio-industry Technology, Jiangsu Ocean University, Lianyungang, 222005 Jiangsu China

**Keywords:** *Cyclina sinensis*, Feces; noninvasive DNA isolation, DNA degradation

## Abstract

**Background:**

To avoid destructive sampling for conservation and genetic assessment, we isolated the DNA of clam *Cyclina sinensis* from their feces. DNA electrophoresis and PCR amplification were used to determine the quality of fecal DNA. And we analyzed the effects of different conditions on the degradation of feces and fecal DNA.

**Results:**

The clear fecal DNA bands were detected by electrophoresis, and PCR amplification using clam fecal DNA as template was effective and reliable, suggesting that clam feces can be used as an ideal material for noninvasive DNA isolation. In addition, by analyzing the effects of different environmental temperatures and soaking times on the degradation of feces and fecal DNA, we found that the optimum temperature was 4 °C. In 15 days, the feces maintained good texture, and the quality of fecal DNA was good. At 28 °C, the feces degraded in 5 days, and the quality of fecal DNA was poor.

**Conclusions:**

The clam feces can be used as an ideal material for noninvasive DNA isolation. Moreover, the quality of fecal DNA is negatively correlated with environmental temperature and soaking time.

## Background

The clam *Cyclina sinensis* is an economically important marine bivalve that is abundant and widely distributed around the maritime coasts of Asia. *C. sinensis* is a kind of eurythermal and euryhaline filter-feeding clam, and its food source mainly includes planktonic microalgae (*Nannochloropsis oculata*, *Chaetoceros muelleri*, *Isochrysis galbana*,etc.) [1, 2] and the remains of organic debris by filtering water and sometimes opepods, facilitating the formation of fecal texture. *C. sinensis* has two hard and symmetrical shells on both sides, and it will quickly close the shells to protect itself from damage when it is stimulated by outside environment. Destructive and nondestructive sampling methods are often applied in scientific researches of clam [3, 4]. The former is conducted by taking parts of specific tissue after the experimental animals are dissected directly, whereas the latter is usually completed by means of a shell opener or a mini electric drill. Nevertheless, sampling using both methods will negatively influence the life of clams, even leading to their death.

Noninvasive sampling is a sampling method for genetic analysis by collecting exfoliated hair, feces, and urine without having to catch, handle, or even observe the animals [5]. It has been widely used in the field of conservation genetics because it is simple and does not harm experimental animals. At present, noninvasive sampling methods are being applied to fish and marine mammals by collecting body surface mucus [6], shedding scales [7], and feces [8, 9]. Among them, feces can be easily collected without disturbing or negatively affecting the normal life of experimental animals. Therefore, feces are potentially valuable research materials in noninvasive sampling. The main component of feces is undigested food residues, where intestinal epithelial cells adhere to when they pass through the intestine. Therefore, mitochondrial and nuclear genomic DNA can be isolated from the remaining epithelial cells in the feces [10]. Fecal molecular biotechnology provides a rapid and dependable way of sampling endangered animals [11–14]. In addition, with the development of molecular biology technology, fecal DNA is extensively used in genetic biology studies for species identification [15–17], individual identification [18–20], sex identification [21–25], population genetic structure [26–28], and genetic diversity evaluation [29]. However, fecal sampling has some problems, such as poor fecal DNA isolation quality and low success rate of PCR amplification [30]. Moreover, no study has performed fecal DNA extraction on invertebrates, especially shellfish. Studies on terrestrial animals have found that fecal DNA degradation occurs with the increase of exposure time [31] and is affected by many other factors, such as light, temperature, and humidity [32, 33]. Compared with those of terrestrial animals, the feces of aquatic animals are more vulnerable due to the external water environment, and their fecal DNA is easier to degrade. Therefore, to obtain good quality shellfish fecal DNA, an improved fecal DNA extraction method should be developed, and the optimal environmental conditions for fecal sampling should be investigated.

In this study, clam feces was used as an experimental material to isolate DNA noninvasively. Moreover, the effects of environmental temperature and soaking time on the degradation of feces and fecal DNA were analyzed. The results can be used as a basis for developing noninvasive DNA isolation technology of shellfish and provide a reference for optimal conditions of fecal sampling, providing technical support for further research on molecular biology and conservation genetics of shellfish.

## Results

### DNA isolation of fresh feces

**Fig. 1:**
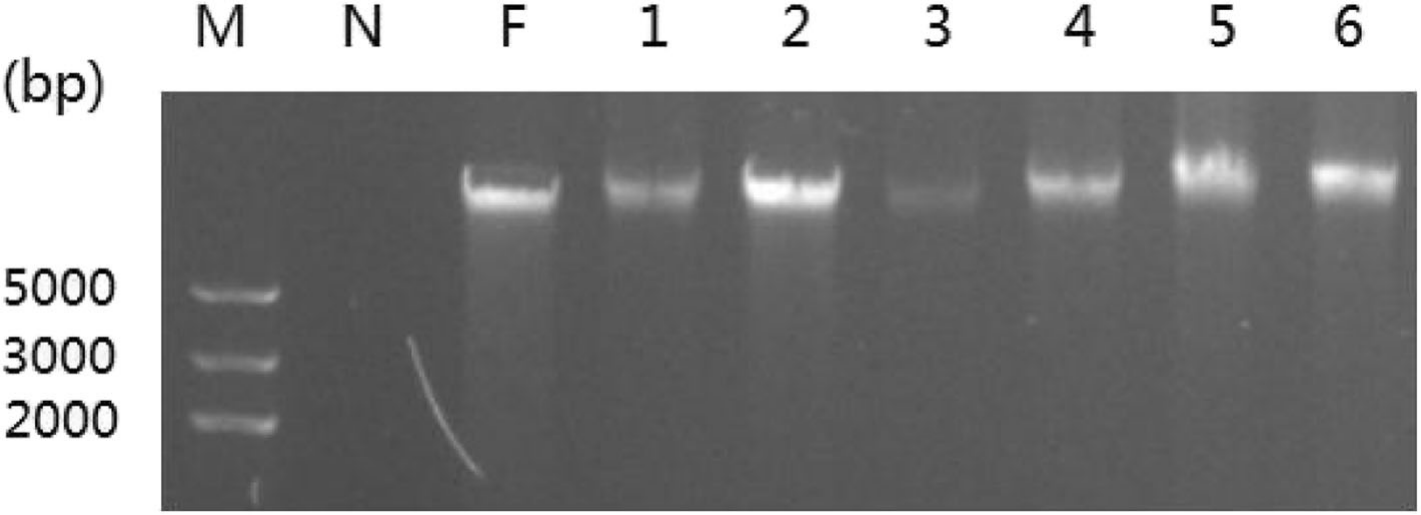
Agarosegel electrophoresis of fecal DNA. Lane M, DNA marker; lane N, negative control; lane F, foot DNA; lanes 1–6, DNA of fresh feces. (N=6).

To determine the quality of fecal DNA, electrophoresis was conducted, and the foot muscle DNA was chosen as the positive control. The results showed that all bands of the fecal DNA were clear but showed a slight tailing phenomenon (Fig. 1 and Additional file 1: Figure S1), which was proved by the results of A260/280 (Table 1). Moreover, the bands of fecal DNA in lanes 2, 4, 5, and 6 were very bright, similar to the foot DNA band (lane F).

**Table 1:** DNA quality of fresh feces (*N* = 6)

Fecal DNA	A260/280	Concentration (ng/μL)
1	1.62	24.2
2	1.79	27.3
3	1.59	20.5
4	1.70	24.3
5	1.67	23.6
6	1.77	25.1

### PCR amplification

**Fig. 2:**
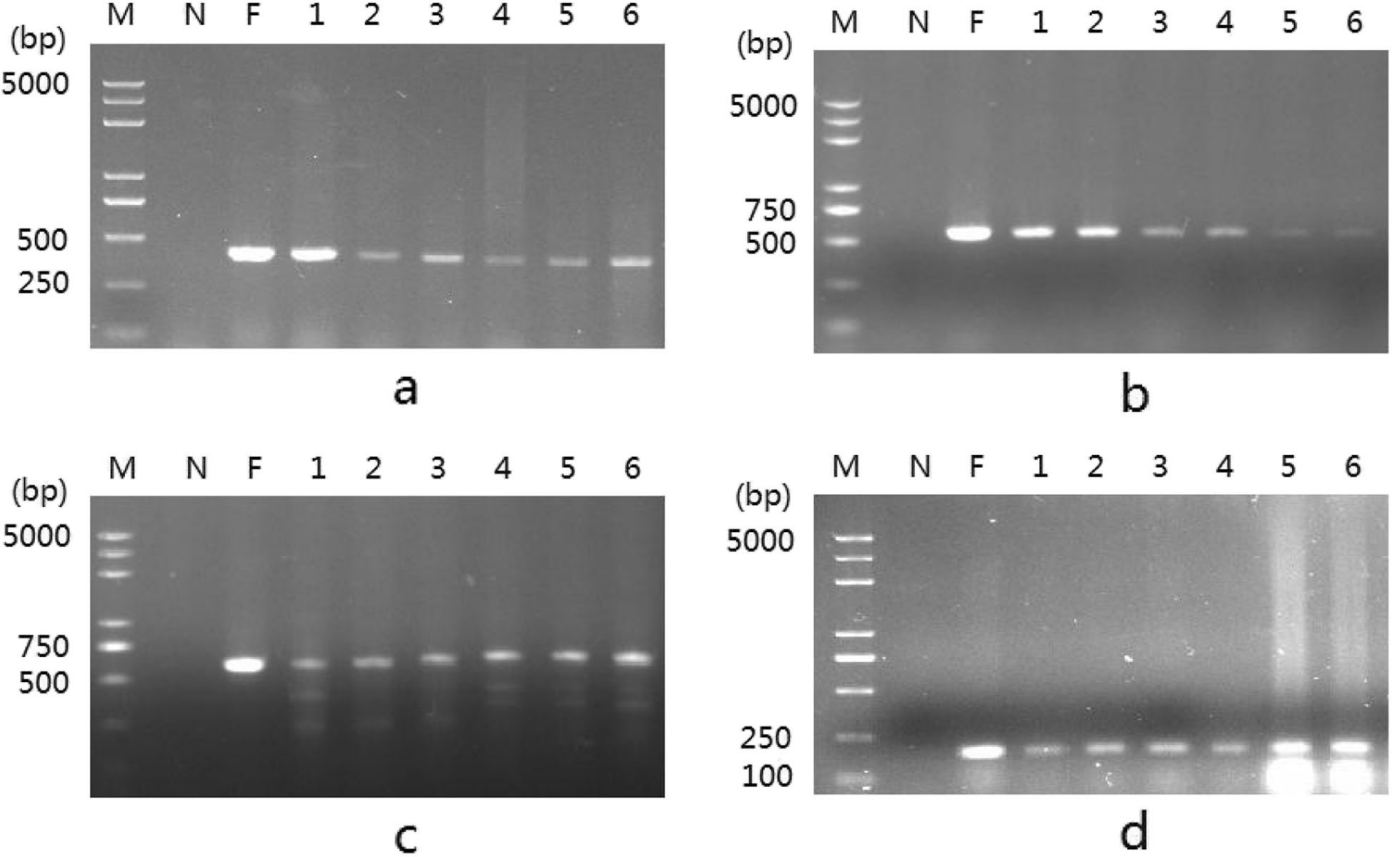
Agarose gel electrophoresis of PCR amplification products. **a**, PCR amplification products using CsCOXI primer; **b**, PCR amplification products using. Cs16S primer; **c**, PCR amplification products using Cs18S primer; **d**, PCR amplification products.using Cspds primer; lane M, DNA marker; lane N, negative control; lane F, PCR amplificationproducts offoot DNA; lanes 1-6,PCR amplification products of fecal DNA(N=6).

To determine the effectiveness of fecal DNA, PCR amplification was conducted using the specific primers designed on the basis of mitochondrial and nuclear genomic DNA of *C. sinensis*. The results revealed that the band size of fecal DNA was the same as that of foot DNA and consistent with the expected length of the target band (Fig. 2 and Additional file 2: Figure S2), which was also proved by the sequencing results.

### Effects of soaking time and environmental temperature on fecal degradation

**Fig. 3:**
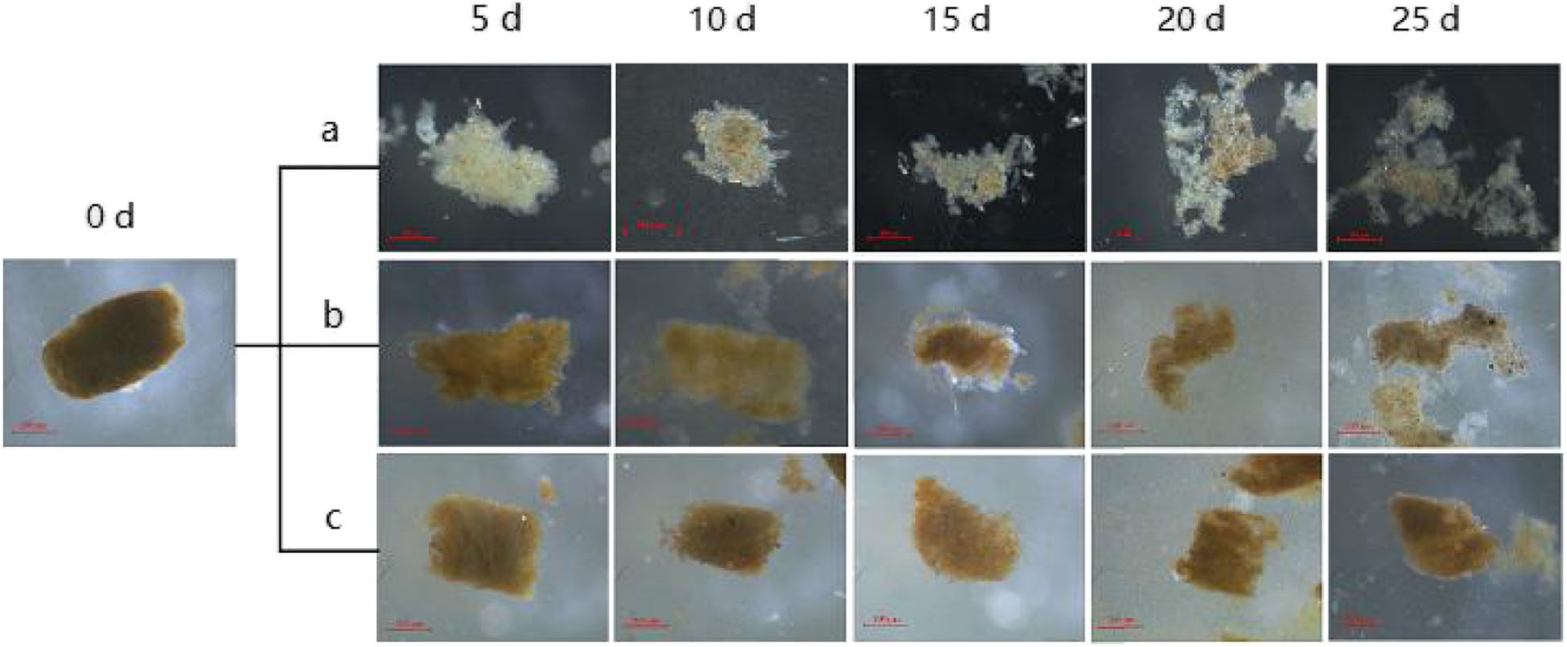
Fecal textures of samples stored at 28 °C (**a**), 15 °C (**b**), and 4 °C (**c**)

Fecal degradation was evaluated by observing changes in fecal texture using a stereoscope. The fecal texture changed over time and was influenced by the environmental temperature. The fresh fecal pellets (0 days) were yellowish green in color and cylindrical. They had a length of 700 μm and diameter of 450 μm (Fig. 3). In fecal samples stored at 28 °C (Fig. 3a), the surface texture became loose at 5 days, with filaments growing abundantly. The filaments grew in large numbers and gradually formed into microbial micelles. More bacteria attached to the microbial micelles, eventually forming bacterial micelles. At 10 and 15 days, the loose feces obviously broke apart, and the local fecal textures were decomposed. At 20 days, the breakage sites increased, and the fecal pellets became looser. At 25 days, the fecal pellets developed into bioflocs framed with filamentous fungi. In fecal samples stored at 15 °C (Fig. 3b), some fecal textures were slightly decomposed on the 10th day. Fecal breakage sites gradually increased at 15–20 days, and large cracks were observed at 25 days. In fecal samples stored at 4 °C (Fig. 3c), the fecal texture was not loose until 10 days and became slightly loose at 15 days. At 20–25 days, some parts of the fecal pellets were slightly decomposed.

### Effects of soaking time and environmental temperature on fecal DNA degradation

**Fig. 4:**
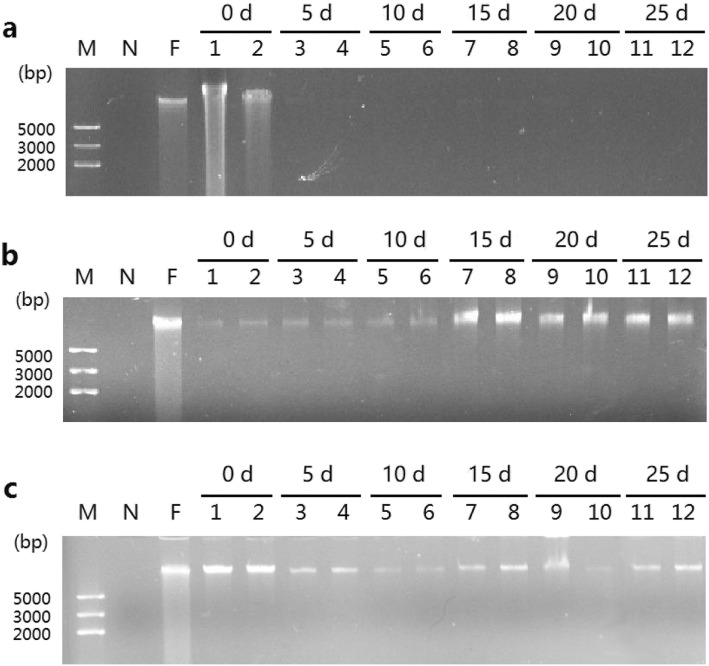
Agarose gel electrophoresis of DNA isolated from clam feces under different soaking times and environmental temperatures. Samples stored at 28°C(**a**), 15°C(**b**), and 4°C(**c**). Lane M, DNA marker; lane N, negative control; lane. F, foot DNA of clam; lanes 1–12, fecal DNA.

Under different soaking times and environmental temperatures, the degradation degree of fecal DNA was determined by agarose gel electrophoresis. At 28 °C, fecal DNA degradation occurred at 5 days after soaking the feces in seawater, but high-quality DNA could still be isolated from few fecal samples (Fig. 4a and Additional file 3: Figure S3a). At 15 and 20 days after soaking, poor-quality DNA was obtained from fecal samples, and serious fecal DNA degradation was observed. At 15 °C, good-quality fecal DNA could still be extracted at 10 days after soaking (Fig. 4b and Additional file 3: Figure S3b); however, the sample degraded to varying degrees after 15 days. At 4 °C, high-quality DNA without tailing phenomenon could still be obtained from fecal samples at 15 days after soaking (Fig. 4c and Additional file 3: Figure S3c).

## Discussion

### Using the modified phenol/chloroform method for fecal DNA isolation and PCR verification

Feces is a very complex mixture of biotic and abiotic components. In this study, DNA was extracted from clam feces, and the quality of fecal DNA was identical to that of foot DNA. As shown in Fig. 2, four specific fragments of mitochondrial and nuclear genomic DNA from *C. sinensis* were amplified by PCR using the fecal DNA as template. The results suggest that the isolation of fecal DNA was successful and reliable, which were proved by sequencing results. Therefore, clam DNA can be nondestructively isolated from feces. However, differences in the quality of fecal DNA were still observed among different fecal samples, which may be due to the quantity variance of intestinal cells adhered to feces. Besides intestinal cells, feces contain undigested food, digestive enzymes, mucus, and other blockers, which affect Taq DNA polymerase activity [34–36]. Furthermore, by extracting fecal DNA from diet-restricted brown bears, Murphy et al. found that the diet has a significant effect on the success rate of PCR amplification using fecal DNA as template [22]. In this study, the success rate of PCR amplification using fecal DNA as template was 100%. This finding can be explained by two factors: (i) *C. sinensis* have special feeding habits (mostly microalgae), which may cause their feces to contain few inhibitors to Taq DNA polymerase. (ii) The modified phenol/chloroform method was effective in isolating DNA from feces. Taken together, the findings indicate that the modified phenol/chloroform method is effective in isolating DNA from feces, and fecal DNA from herbivorous animals can be used as a template for PCR amplification, which is also supported by the findings of Zhang et al.’s study on fecal DNA of pandas [37].

### Degradation of feces and fecal DNA under different soaking times and environmental temperatures

Bioflocs are composed of microorganisms, protozoa, algae, filamentous bacteria, and organic matter in water [38]. With the decomposition of feces into flocs, the microbial community structure was altered. In the process of degradation, the structure of fecal pellets became loose, which increased the contact area with seawater, thereby increasing the attachments for protozoa and leading to a looser structure [39]. As shown in Fig. 3, differences were observed in the rate of fecal texture changes at different temperatures. The degradation rate at 28 °C was significantly higher than that at 15 °C and 4 °C after the same soaking time, which was probably due to the high temperature (28 °C) appropriate for the growth and reproduction of microorganisms in feces and improvement of the activity of fecal degradation-related enzymes [40]. Moreover, fecal degradation was often accompanied by fecal DNA degradation (Fig. 4), and the degree of fecal DNA degradation was also affected by the environmental temperature. Similar phenomena were observed in feces of other animals. The quality of DNA isolated from feces of *Canis lupus* in winter remained significantly higher than that in summer [41]. Moreover, the quality of fecal DNA from ape was negatively correlated with fecal environmental temperature [42]. These findings suggest that fecal sampling should be conducted in seasons with low temperature to obtain good-quality fecal DNA [43–45].

The degradation of feces and fecal DNA was also affected by soaking time in seawater. The longer the soaking time, the more serious the degradation (Fig. 4). Several studies suggest that fecal DNA degradation is affected by water. The rate of fecal DNA degradation is significantly accelerated by rain wash [46], and removing water from feces can essentially prevent the activation of nuclease in feces [47]. Moreover, *Cyclina sinensis* is a kind of marine shellfish, and its feces are soaked in seawater. Seawater is a very complicated multicomponent aqueous solution containing various organic, inorganic, dissolved, and suspended substances, which may be the reason for the degradation of feces and fecal DNA from clam. Therefore, the fresher the fecal samples collected, the higher the quality of DNA [48].

## Conclusions

In this study, clam feces were used as experimental material to isolate DNA noninvasively. The isolation of fecal DNA was found to be successful and reliable by PCR amplification. The effects of different environmental temperatures and soaking times on the degradation of feces and fecal DNA were investigated. The results suggest that fresh fecal samples stored at low environmental temperature (~ 4 °C) were beneficial to the isolation of fecal DNA with good quality. This study provides technical support for further molecular biology research and conservation genetics research of shellfish.

## Methods

### Sampling and processing

Healthy clams *C. sinensis* were collected from a clam farm in Jiangsu, China. They were cultured in seawater for two weeks at room temperature and fed with 0.005 g/mL of *Chlorella* once a day. Natural seawater was filtered with a double-layer 500-mesh sieve after precipitation, disinfection, and aeration for culturing clams and replaced once a day.

Forty-eight healthy clams (body weight, 10.09 ± 2.81 g; shell length, 3.01 ± 0.38 cm) were randomly selected and divided into 12 parallel groups (labeled 1 to 12), each groups containing four clams. Feeding was withheld for 2 days in continuously oxygenated seawater. Thereafter, the clams were fed with 6 × 10^5^ cells/L of *Chlorella* until waste matter (feces) was completely expelled. During this period, clam defecation was observed every 2 h. The feces were collected from the bottom of the beaker using siphon method.

### DNA isolation

Total DNA was isolated from clam feces and foot tissue (used as positive control). Fecal DNA isolation was performed using the phenol/chloroform method in accordance with a previous study of Sambrook [49] with some modifications as follows:

1. Place the feces on a 200-mesh silk screen, and wash it slowly with double-distilled water (ddH_2_O) to remove impurities on the fecal surface.
2. Transfer each 200 mg fecal sample into a new 1.5 mL Eppendorf tube.
3. Add 100 μL ddH_2_O, blow the feces repeatedly with a straw to make it homogenate, and then vortex fully.
4. Centrifuge for 3 min at 800×g, and then transfer the supernatant into a new 1.5 mL tube. Add 400 μL of 10% SDS and 10 μL of proteinase K into each tube, and then vortex fully.
5. Incubate the tubes for 1 h in a 65 °C water bath with occasional shaking (~ 10 min).
6. Add 10 μL of 20 mg/mL RNase, and incubate the tubes in a 37 °C water bath for 10 min. Centrifuge for 3 min at 12,000×g, and then transfer the supernatant into a new 1.5 mL tube.
7. Add an equal volume of ice-cold Tris-saturated phenol (pH 7.9), and mix upside down and store at room temperature for 5 min.
8. Centrifuge at 12,000×g for 12 min, and then transfer the supernatant into a new 1.5 mL tube.
9. Add an equal volume of chloroform, and mix upside down. Centrifuge at 12,000×g for 10 min, and then transfer the supernatant into a new 1.5 mL tube.
10. Add an equal volume of isopropanol, mix upside down, and store at room temperature for 3 min. Centrifuge at 12,000×g for 12 min, and then remove the supernatant completely.
11. Wash the DNA pellet twice with 1 mL ice-cold 70% ethanol.
12. Air dry.
13. Resuspend the DNA pellet in 30 μL of TE buffer, and then store at − 40 °C before use.

### Primer design and PCR amplification

To determine DNA quality, PCR amplification was conducted with primers designed on the basis of mitochondrial and nuclear genomic DNA sequences. The sequences of mitochondrial (COXI and 16S rRNA) and nuclear genomic DNA (18S rRNA and partial sequence of nuclear DNA) were retrieved and downloaded from NCBI (https://www.ncbi.nlm.nih.gov/). PCR primers were designed by Primer Premier 5.0 software and are shown in Table 2. PCR amplification was conducted in a 15 μL reaction volume, containing 1.0 μL of DNA template, 0.2 μL of Taq (Takara, Dalian, China), 0.8 μL of primers (including forward and reverse primers), 1.0 μL of dNTPs, 1.5 μL of 10× buffer, and 10.5 μL of ddH_2_O. The PCR amplification procedure was conducted as follows: initial denaturation at 95 °C for 5 min, followed by 30 cycles of denaturation at 94 °C for 1 min, annealing for 30 s, extension at 72 °C for 30 s, and final extension at 72 °C for 10 min. The PCR amplification products were detected by 1.5% agarose gel electrophoresis and captured with a gel imaging system (Universal Hood II, Bio-Rad, USA). The purified PCR products were sequenced by Shanghai Map Biotech Co., Ltd. The sequencing results were checked by Chromas software and blasted by BLAST online software (https://blast.ncbi.nlm.nih.gov/Blast.cgi).

**Table 2:** Primers and sequences

Primer	Sequence (5′–3′)	Source	Gene ID	Product size/bp
CsCOXI	F:TGGTGGTTTAACTGGTGTTGTT	Mitochondrial DNA	26,898,108	404
	R:AAAACACCAAACCACGCTGAG	from *C. sinensis*		
Cs16S	F:GATCGTACCTGCCCTGTGAT	Mitochondrial DNA	26,898,076	548
	R:ACCACTCTAGCTTACGCCGA	from *C. sinensis*		
Cs18S	F:TGCGTTCAAGGTGTCGATGT	Nuclear genomic	unregistered	581
	R:GGGGCCGACATGAAATGAAA	DNA from *C. sinensis*		
Cspds	F: ACTTCAGAATTCAGAATTCAG	Nuclear genomic	unregistered	187
	R: GTCACGCACAATGTAACG	DNA from *C. sinensis*		

### Effects of soaking time and environmental temperature on the degradation of feces and fecal DNA

To explore the effects of environmental temperature and soaking time on fecal texture changes and fecal DNA degradation, the fecal samples were collected immediately after the clams defecated. They were then soaked in clean seawater and stored at 28 °C, 15 °C, and 4 °C. To observe fecal texture changes, the fecal samples were placed on clean slides, observed, and photographed with a stereoscope (Nikon SME 1500, Nikon, Japan) at 0, 5, 10, 15, 20, and 25 days after soaking in seawater. Fecal DNA isolation was conducted using the modified phenol/chloroform method mentioned above, and the fecal DNA quality was determined by Ultramicro Nucleic Acid Analyser (Eppendorf BioPhotometer® D30, Eppendorf, Germany), electrophoresis, and PCR amplification.

### Data analysis

The DNA purity was confirmed by Ultramicro Nucleic Acid Analyser (Eppendorf BioPhotometer® D30, Eppendorf, Germany). The DNA and PCR amplification products were detected by 1 and 1.5% agarose gel electrophoresis respectively, and the gel images were observed and captured with a gel imaging system (Universal Hood II, Bio-Rad, America).

## Supplementary information

**Additional file 1: Figure S1.** Agarose gel electrophoresis of fecal DNA. Lane M, DNA marker; lane N, negative control; lane F, foot DNA; lanes 1–20, DNA of fresh feces (*N* = 20).

**Additional file 2: Figure S2.** Agarose gel electrophoresis of PCR amplification products Lane M, DNA marker; lane N, negative control; lane F, PCR amplification products of foot DNA; lanes 1–20, PCR amplification products of fecal DNA (*N* = 20).

**Additional file 3: Figure S3.** Agarose gel electrophoresis of PCR amplification products from clam feces under different soaking times and environmental temperatures. Samples stored at 28 °C (a), 15 °C (b), and 4 °C (c). Lane M, DNA marker; lane N, negative control; lane F, foot DNA of clam; lanes 1–12, fecal DNA.

## Data Availability

The datasets analyzed during the current study are available from the corresponding author on reasonable request.

## Abbreviations

*C. sinensis*: *Cyclina sinensis*
PCR: Polymerase chain reaction
rDNA: Ribosomal DNA
rRNA: Ribosomal RNA
