# Supplementary material for: Fecal DNA isolation and degradation in clam Cyclina sinensis: noninvasive DNA isolation for conservation and genetic assessment
Source: BMC Biotechnol. 2019 Dec 19;19:99. doi: 10.1186/s12896-019-0595-6 (PMC6923993; doi:10.1186/s12896-019-0595-6)
Supplement: Supplementary file 1 — Additional file 1: Figure S1. Agarose gel electrophoresis of fecal DNA. Lane M, DNA marker; lane N, negative control; lane F, foot DNA; lanes 1–20, DNA of fresh feces (N = 20). [file 12896_2019_595_MOESM1_ESM.pdf]

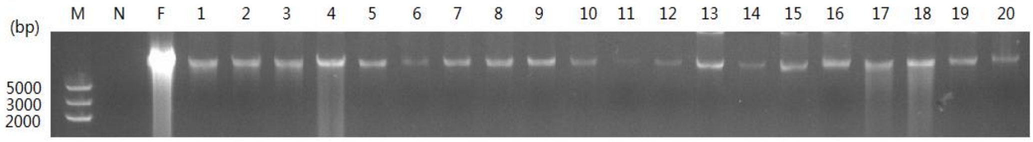

Fig. S1 Agarose gel electrophoresis of fecal DNA.

Lane M, DNA marker; lane N, negative control; lane F, foot DNA; lanes 1–20, DNA of fresh feces (N=20).
