# Supplementary material for: Fecal DNA isolation and degradation in clam Cyclina sinensis: noninvasive DNA isolation for conservation and genetic assessment
Source: BMC Biotechnol. 2019 Dec 19;19:99. doi: 10.1186/s12896-019-0595-6 (PMC6923993; doi:10.1186/s12896-019-0595-6)
Supplement: Supplementary file 2 — Additional file 2: Figure S2. Agarose gel electrophoresis of PCR amplification products Lane M, DNA marker; lane N, negative control; lane F, PCR amplification products of foot DNA; lanes 1–20, PCR amplification products of fecal DNA (N = 20). [file 12896_2019_595_MOESM2_ESM.pdf]

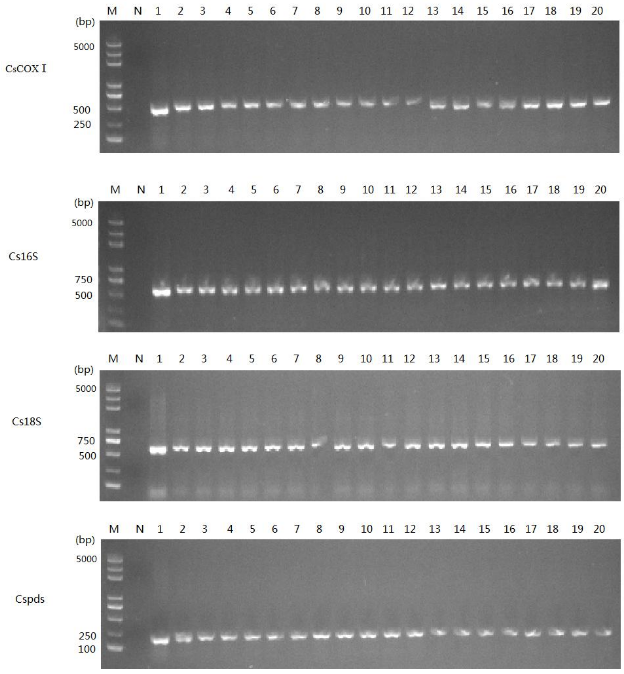

Fig. S2 Agarose gel electrophoresis of PCR amplification products

Lane M, DNA marker; lane N, negative control; lane F, PCR amplification products of foot DNA; lanes 1-20, PCR amplification products of fecal DNA (N=20).
